# Interrupting the Cycle: Association of Parental Stress and Child/Youth Psychotropic Medication Nonadherence

**DOI:** 10.1007/s10578-022-01448-y

**Published:** 2022-10-28

**Authors:** Laura Theall, Ajit Ninan, Kim Arbeau, Jessica Mannone, Shannon L. Stewart

**Affiliations:** 1 Applied Research & Education, Child and Parent Resource Institute, 600 Sanatorium Road, N6H 3W7 London, ON Canada; 2 Division of Child and Adolescent Psychiatry, Western University, Parkwood Institute Mental Health Care Building, F4-430, N6C 0A7 London, ON Canada; 3 Faculty of Education, Western University, 1151 Richmond Street, N6A 3K7 London, ON Canada

**Keywords:** Psychotropic medication, Nonadherence, Family, Children and youth, InterRAI

## Abstract

Efficacy of psychotropic medication depends in large part on successful adherence to prescribed regimens. This study investigated child/youth nonadherence in relation to family dynamics and informal support. The participants were 10,225 children and youth prescribed psychotropic medication and receiving services from 50 Ontario mental health agencies, assessed with the interRAI™ Child and Youth Mental Health (ChYMH) and ChYMH-Developmental Disability (ChYMH-DD) tools. Findings suggest a cycle of parental stress and child/youth medication nonadherence possibly leading to or even perpetuated by worsening psychiatric symptoms. Informal supports do not appear to moderate this cycle. While the present data cannot speak to causes of medication nonadherence in children/youth or where the cycle begins, the results are consistent with the extant literature calling for attention to parental wellbeing to support children/youth for optimal therapeutic benefits. Understanding home dynamics related to nonadherence can assist care planning that engages the family to achieve best possible child/youth outcomes.

## Introduction

When used properly, psychotropic medication can be an effective component in treating severe emotional and behavioural disorders in children and youth [1]. Effective intervention at an early life stage can potentially avoid a life-long trajectory of mental health problems [2, 3]. Not taking medication as prescribed, known as nonadherence, can lead to poor treatment outcomes and potential rehospitalization [4, 5], or possible overdose [6].

Factors known to impede medication adherence include the child/youth experiencing adverse side effects, forgetting to take medication, and feeling better [7]. Also, external factors such as negative parental attitudes about medication and lack of support from family members are related to medication nonadherence in children and youth [8–10]. Previous research has found that absence of parental assistance with medications predicted nonadherence in children/youth [11, 12], and parents’ perception of medication can impact their child/youth’s opinion of prescribed psychotropics [13, 14]. Parent personality type can also play a role, with high conscientiousness associated with positive communication with service providers and involvement in the medication routine facilitating child/youth medication adherence. Conversely, high parental neuroticism has been found to be associated with child/youth nonadherence and disengagement from health-related decisions [4]. Other mechanisms by which the family influences adherence have been relatively unexplored within child populations.

In adult populations informal sources of support from extended family and friends have been found to have a positive impact on taking medications as prescribed [15, 16], and lack of social support has been associated with medication nonadherence [17]. While the family is certainly a core source of support for children/youth and an established factor in medication adherence, there is a paucity of research investigating whether informal support available to the family by extension is associated with child/youth medication adherence. Availability of informal support to the family of young people is often overlooked when the direct focus of intervention is assisting the child/youth [18].

Given the limited extant literature, the primary objective of the present study was to explore the relation between medication nonadherence for children/youth with complex needs and family dynamics. Specifically, this objective was designed to further understand if parents who are stressed, overwhelmed with the child/youth’s condition, and critical or hostile towards the young person have a higher likelihood of their child/youth’s nonadherence to psychotropic medication. A second objective was to extend the research on the association between informal support and adult medication adherence to child/youth populations by investigating whether families who lack support from extended family and friends for life challenges have children/youth who are nonadherent to prescribed psychotropic medications.

## Method

### Participants

Clinical data collected from 2012 to early 2020 was used from 10,225 children and youth ages 4–18 years old (*M*_*age*_=12.72, *SD* = 3.32; 58.1% male, 41.6% female, 0.3% other) who were prescribed psychotropic medication and receiving services from 50 mental health agencies across Ontario. The participants were assessed using the interRAI™ Child and Youth Mental Health (ChYMH) or the interRAI Child and Youth Mental Health – Developmental Disability (ChYMH-DD) instrument at the agencies where they were receiving services for emotional and behavioural challenges (including developmental disabilities, autism, and mental health concerns; 92% outpatient, 8% inpatient). Assessment type in the current study was the initial assessment which typically occurs at intake. Ethics clearance for secondary data analyses was obtained from Western University (REB #106415).

*Psychiatric presentation.* The reason for referral to mental health services was danger to self and/or others for 53% of the children and youth: 15% danger to self, 19% danger to others, and 19% were referred for danger to both themselves and others. Common provisional psychiatric diagnoses included Attention Deficit Hyperactivity Disorder (ADHD, 54%), anxiety disorders (43%), Disruptive Behaviour Disorder (24%), learning and communication disorders (24%), mood disorders (22%), Autism Spectrum Disorder (ASD, 13%), and Reactive Attachment Disorder (RAD, 3%). Only 15% of children/youth did not hold any of these selected diagnoses at the time of assessment. While 25% of participants held one of the diagnoses listed above, complexity of psychiatric condition was evident for 60% of the children/youth who held two or more of these. It should be noted that since this was at initial assessment diagnoses likely altered for some children/youth during their consultations/assessments with clinicians/physicians at the agencies.

*Family structure.* Most of the participants lived with their parent(s) (89%). There were 57% in households with two adults, and one quarter (25%) in single-adult households. Other living situations included 14% with more than two adults, 4% not reported, and 0.1% no adults in the home. There were 43% of participants with parents who were married or had partners, and 27% with separated or divorced parents. Legal guardianship was both parents for 56%, mother only for 28%, and father only for 4%. Other types of guardianship included 6% other relative or non-relative, 5% child protection agency or public guardian, and 0.4% youth responsible for self. Socio-economic and race-based data were not collected.

### Measures

*interRAI™ Child and Youth Mental Health (ChYMH)* [19] *and ChYMH-Developmental Disability (ChYMH-DD)* [20]. The ChYMH and ChYMH-DD are multi-source assessment instruments that are part of a comprehensive health information system inclusive of the lifespan [21]. Children and youth with neurotypical development are assessed using the ChYMH, and the ChYMH-DD is adapted for children and youth with neurodevelopmental differences. Both tools are used to assess strengths and needs for initial triaging, care planning, and to evaluate outcomes of services [22]. In the current sample, 94% of the data came from the ChYMH and 6% from the ChYMH-DD. The ChYMH and ChYMH-DD are completed by trained assessors using all available sources (e.g., the child/youth, caregivers, service providers, clinical records). A web-based software system is used to securely store the data and calculate results. All personal identifiers were removed before access to the data was made available for analysis.

Within the ChYMH and ChYMH-DD are algorithms that trigger best practice care planning guidelines called Collaborative Action Plans (CAPs) developed in collaboration with subject matter experts [23, 24]. The current study used the triggering algorithm of the Medication Adherence CAP as the dependent variable which flags children/youth who are currently or at high risk for not taking psychotropic medication as prescribed (e.g., was adherent less than 80% of the time, refused to take medications, and/or needed assistance with medication use but whose parent did not assist). The CAP also provides guidelines for clinicians to assist in improving adherence to prescribed treatment schedules. In the current sample, 2154 (21%) of young persons were flagged for nonadherence by the Medication Adherence CAP. Information on the specific kinds of psychotropic medications prescribed was not available in the dataset. The adherent and nonadherent groups were similar in psychiatric complexity as the proportion of participants with two or more of the afore mentioned provisional psychiatric diagnoses flagged for nonadherence was 57%, and 61% of children/youth in the adherent group held two or more diagnoses.

### Statistical Analyses

Preliminary chi-square analysis indicated that significantly more youth, 27% (*n* = 1771; ages 12–18 years), were flagged for medication nonadherence than younger children at only 10% (*n* = 383; ages 4–11 years) [*X*^2^(1, 10,225) = 379.74, *p* = .000]. Therefore, the analyses considered children and youth separately.

Multiple logistic regression analyses were conducted to predict medication nonadherence for children and youth from select variables. The independent variables included the following parental/family difficulties: parental stress, feeling of being overwhelmed, hostility toward the child/youth, and lack of informal support. Parental stress was indicated using the Caregiver Distress CAP which flags high levels of parental difficulties that may be impacting the mental health of the young person (i.e., parent demonstrates two or more of the following indicators: unwilling to continue in caring activities, feelings of distress, anger, depression or other mental health issue, substance abuse or other addiction, parental conflict or custody dispute, financial difficulties, or other major life stressors in the past 3 months) [23, 24]. Individual items were used from the interRAI tools to measure feeling overwhelmed (i.e., Family feels overwhelmed by the child/youth’s condition) and hostility (i.e., Family is persistently hostile or critical of the child/youth). Trained assessors scored each item using the response options “no”, “yes”, or “not applicable” if the child/youth had no parent or family. The Informal Support CAP was used to flag families who need but lack support from informal sources such as extended family or friends to help with life challenges. Specifically, the algorithm for the Informal Support CAP identified whether the family needed but had no support in two or more of the following areas: emotional support, babysitting or respite, help in crisis situations, or help with financial problems [23].

## Results

As shown in Table 1, results indicated that high parental stress was a significant predictor of medication nonadherence for children and youth. The items indicative of the family feeling overwhelmed (i.e., Family feels overwhelmed by the child/youth’s condition) and exhibiting hostility (i.e., Family is persistently hostile or critical of the child/youth) also predicted nonadherence for youth, but not younger children. Lastly, whether the family had support from informal sources for life challenges did not predict nonadherence in children or youth.

**Table 1:** Predicting child and youth medication nonadherence from family dynamics and informal support

Variable	Age Group	*B*	*p* value
Caregiver distress	Children	*B* = 0.43, Wald = 12.96, Exp(*B*) = 1.53	*p* = .000
	Youth	*B* = 0.12, Wald = 3.87, Exp(*B*) = 1.30	*p* = .049
Family feels overwhelmed by child/youth’s condition	Children	*B* = 0.10, Wald = 0.72, Exp(*B*) = 1.11	*p* = .395
	Youth	*B* = -0.23., Wald = 13.62, Exp(*B*) = 0.80	*p* = .000
Family is persistently hostile/critical of child/youth	Children	*B* = 0.26, Wald = 2.80, Exp(*B*) = 1.30	*p* = .094
	Youth	*B* = 0.52, Wald = 53.37, Exp(*B*) = 1.69	*p* = .000
Informal support to the family	Children	*B* = 0.31, Wald = 0.61, Exp(*B*) = 1.03	*p* = .805
	Youth	*B* = -0.09, Wald = 1.47, Exp(*B*) = 0.92	*p* = .225

### Post-hoc Investigation

The significant relation between parental stress and child/youth medication nonadherence prompted a post-hoc research question: Did those children and youth who were nonadherent to medication with stressed parents exhibit worsening psychiatric symptoms? We hypothesized that problem behaviours may increase in the absence of medication adherence, which could impact parental stress levels.

### Post-hoc Analyses

Chi-square analyses were conducted for children and youth separately. Selecting only those nonadherent to medication, high and low parental stress as indicated by the Caregiver Distress CAP was investigated using chi-square analyses in relation to the following variable from the same dataset: Change in severity or frequency of psychiatric symptoms compared to 30 days ago, or since last assessment if less than 30 days ago (scored as: (1) Deterioration; (2) No change; (3) Improvement; (4) Marked improvement). Improvement and marked improvement were collapsed into a single Improvement category. To record a response to this item, trained assessors were instructed to consider symptom frequency and intensity as part of the assessment by reviewing the clinical record and care plan, and consulting with other involved clinicians or the referral source.

### Post-hoc Results

**Fig. 1:**
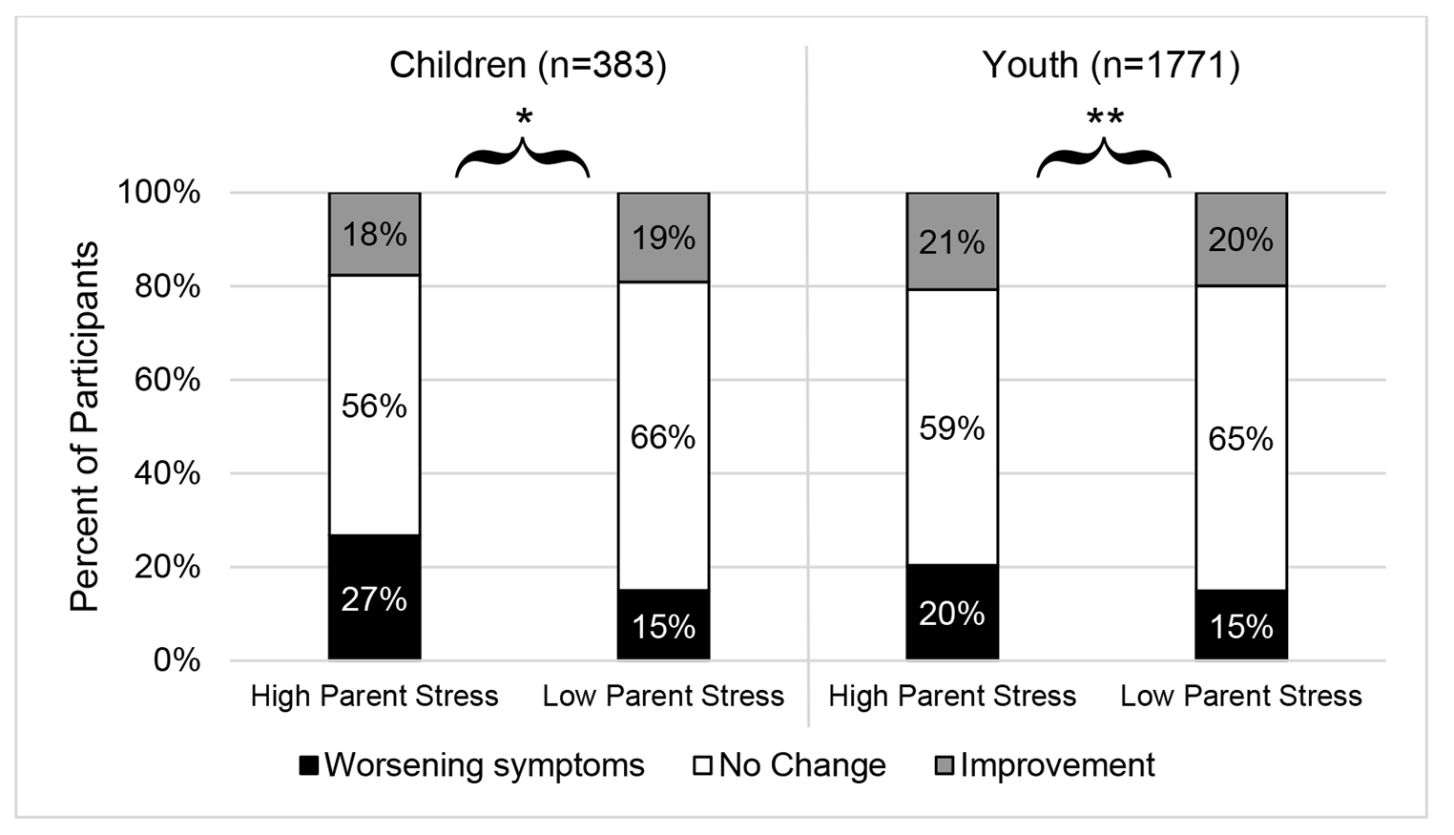
Post-hoc investigation of the percent of nonadherent children and youth with parents with high or low stress who experienced worsening psychiatric symptoms, no change, or improvement in the previous month. Note. * p < .05; ** *p* < .01

Significant results indicated differences in stability of symptoms for nonadherent children and youth depending on parental stress level: Children *X*^2^(2, 383) = 7.74, *p* = .02; youth *X*^2^(2, 1771) = 10.60, *p* = .005. As shown in Figs. 1 and 12% more nonadherent children and 5% more nonadherent youth with parents flagged for high levels of stress experienced worsening psychiatric symptoms within the past month compared to nonadherent children and youth of low stress parents.

## Discussion

**Fig. 2:**
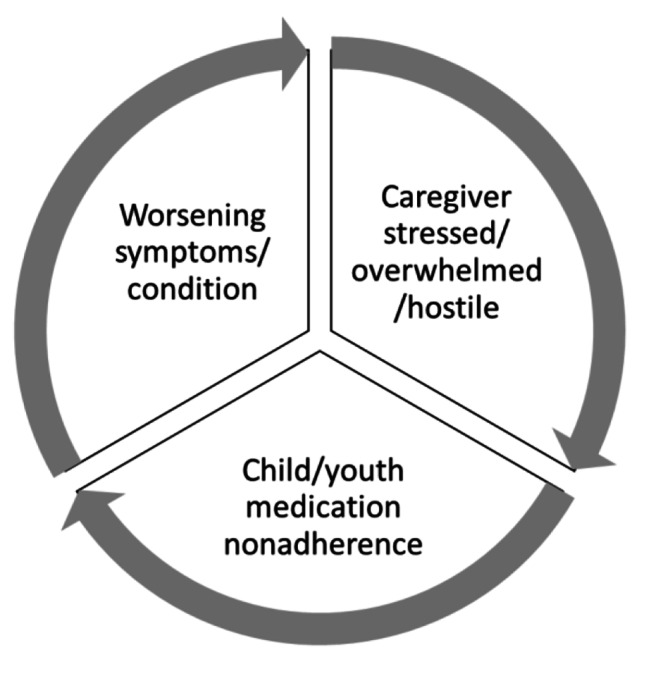
Potential cycle of medication nonadherence and family dynamics

Children/youth who were nonadherent to psychotropic medication tended to have parents who were stressed and thus may not be emotionally available to actively support the prescribed treatment plan [4]. Additionally, manifestations of stress including overwhelmed feelings due to the youth’s condition and family hostility towards youth (but not younger children) predicted nonadherence as well. Examining changes in psychiatric symptoms, the current study showed some evidence of worsening symptoms for nonadherent children and youth with stressed parents. Building on the literature that the family in general, and parents in particular, can influence medication adherence, the current study identified a potential cycle that emphasizes the disadvantages of medication nonadherence as it interferes with therapeutic benefits and family functioning (see Fig. 2).

While the present data cannot speak to the causes of medication nonadherence in children/youth or where the cycle begins, the results are consistent with the extant literature calling for attention to parental wellbeing to support children/youth for optimal therapeutic benefits [25]. The younger a child is, the more dependent they are on parental administration of prescribed medications [26]. Therefore, this childhood dependency may strongly impact the cycle. Older children/youth may be attributed with greater independence to self-administer psychotropic medications [27]. However, if there is a lack of parent involvement, potentially due to the family dynamic variables examined in the current study, this may negatively impact medication adherence given the relative absence of oversight or encouragement [11]. Parenting a child/youth with severe emotional and behavioural challenges brings unique stressors within the household and can generate tensions within family dynamics [18, 28, 29]. Further, parental stress and hostility have been found to impact child/youth behaviours and wellbeing [25]. Nonadherence to prescribed psychotropic medication can lead to worsening mental health symptoms or conditions for children/youth, and more severe symptoms are associated with greater risk for nonadherence [30]. With worsening mental health symptoms, problem behaviours can escalate, increasing stress and overwhelming parents further which, in turn, predicts medication nonadherence based on the current data. Interrupting this cycle with evidence-informed care planning that involves the family is essential [23, 24].

Ways to improve medication adherence include involving all family members in each stage of care planning for collaborative decision-making with the child/youth to incorporate their goals [31]. In addition, involving the family in discharge planning from mental health services can help to maintain treatment gains and prevent readmission [32]. Improving parent and service provider communication could facilitate adherence to the child/youth’s medication regimen [4, 33, 34]. Adherence could also be enhanced by elevating older youth’s levels of autonomy and providing them with a sense of responsibility within their medication regimen [14, 33]. Engaging the family through interventions that support the child/youth’s medication routine, including effective nonjudgmental communication, fosters medication adherence and empowers individual motivation with the family’s encouragement [34, 35]. Interventions designed to promote adherence have also been found to have a positive impact on family dynamics by way of improving communication and problem-solving [36].

The present study found an association between parental stress and child/youth nonadherence, suggesting that implementing effective supports to manage parental stress may have a positive effect on child/youth medication adherence. While informal supports may be effective for alleviating some parental stress, there was an absence of association between lack of informal support available to parents and child/youth medication nonadherence. Formal supports such as peer support groups, professional consultation, and prescribed respite care may be more beneficial in managing family challenges and parental stress as the complexity of concerns may be beyond the capabilities of what can be supported by extended family and friends. Past research has found that while having informal supports reduced inflammation related to stress in parents of children/youth with neurodevelopmental challenges, formal supports had a greater effect on improving parent self-reported health and somatic symptoms [37]. Formal peer consultant services have been found to help parents to cope with stressors associated with their child/youth’s inpatient admission [18]. Virtual sources of informal support such as social media and online forums were not explored in the current study but may be beneficial to parents’ emotional wellbeing [38], possibly thereby promoting parents’ involvement in their child/youth’s medication routine.

There were 27% of participants who had separated or divorced parents. It is important for both parents in separate homes to be involved and support the child/youth’s medication schedule. Whether parents had mental health issues was included in the caregiver distress variable in the current study. There were 47% of parents in the nonadherent group reported to have their own developmental/mental health issues. Semahegn and colleagues reported that 49% of adults who had major psychiatric illnesses were not adherent to their own medications [17]. It may be difficult for parents who themselves struggle to adhere to a medication regime to support children/youth who are also prescribed psychotropic medications.

## Limitations

A limitation of the current study is the absence of socio-economic data and how this might relate to family challenges. For example, two-parent households have been found to predict better medication adherence for children and youth with anxiety [10], but whether certain socio-economic advantages or supportive family dynamics are at work in that association remains unknown. In the current study, approximately half of children/youth lived in two-adult households, but whether this translated directly into the number of parents in the home and/or socio-economic stability is unidentified in the data. A second potential limitation is that adherence was measured by subjective means of self report rather than objective means such as pill counts or medication levels in the bloodstream. A recent meta-analysis helps to alleviate this concern as subjective and objective measures of medication adherence were found to be comparable [39]. Lastly, it is important to note that the data for this study was collected prior to the COVID-19 pandemic (up to January 2020), which represents a timeframe before unique stressors caused by pandemic-related restrictions. Pandemic factors possibly facilitating or impeding adherence to psychotropic medication should be explored by future research, such as any impact of increased time spent within the home due to school/work/recreation closures, changes in access to mental health care from in-person to virtual appointments, family social isolation, job loss, increased financial constraints, illness, and other pandemic-related stressors for families.

## Summary

Family dynamics have an important role in medication nonadherence in children and youth, specifically parental stress and the parent and youth relationship. The current study indicates a need to interrupt a cycle of child/youth nonadherence, worsening mental health conditions and/or behavioural symptoms, and parental stress with care planning that involves the family, attends to parental wellbeing, and empowers older youth in their own care.
